# A New Classification System for IgG4 Autoantibodies

**DOI:** 10.3389/fimmu.2018.00097

**Published:** 2018-02-12

**Authors:** Inga Koneczny

**Affiliations:** ^1^Institute of Neurology, Medical University of Vienna, Vienna, Austria

**Keywords:** IgG4 autoimmunity, IgG4, autoimmunity, neuronal autoantibodies, muscle-specific kinase myasthenia gravis, thrombotic thrombocytopenic purpura, pemphigus, IgG4-related disease

## Abstract

IgG4 autoimmune diseases are characterized by the presence of antigen-specific autoantibodies of the IgG4 subclass and contain well-characterized diseases such as muscle-specific kinase myasthenia gravis, pemphigus, and thrombotic thrombocytopenic purpura. In recent years, several new diseases were identified, and by now 14 antigens targeted by IgG4 autoantibodies have been described. The IgG4 subclass is considered immunologically inert and functionally monovalent due to structural differences compared to other IgG subclasses. IgG4 usually arises after chronic exposure to antigen and competes with other antibody species, thus “blocking” their pathogenic effector mechanisms. Accordingly, in the context of IgG4 autoimmunity, the pathogenicity of IgG4 is associated with blocking of enzymatic activity or protein–protein interactions of the target antigen. Pathogenicity of IgG4 autoantibodies has not yet been systematically analyzed in IgG4 autoimmune diseases. Here, we establish a modified classification system based on Witebsky’s postulates to determine IgG4 pathogenicity in IgG4 autoimmune diseases, review characteristics and pathogenic mechanisms of IgG4 in these disorders, and also investigate the contribution of other antibody entities to pathophysiology by additional mechanisms. As a result, three classes of IgG4 autoimmune diseases emerge: class I where IgG4 pathogenicity is validated by the use of subclass-specific autoantibodies in animal models and/or *in vitro* models of pathogenicity; class II where IgG4 pathogenicity is highly suspected but lack validation by the use of subclass specific antibodies in *in vitro* models of pathogenicity or animal models; and class III with insufficient data or a pathogenic mechanism associated with multivalent antigen binding. Five out of the 14 IgG4 antigens were validated as class I, five as class II, and four as class III. Antibodies of other IgG subclasses or immunoglobulin classes were present in several diseases and could contribute additional pathogenic mechanisms.

## Introduction

Autoantibodies are key pathogenic players in a wide range of autoimmune diseases. Immunoglobulins (Ig) of IgG or IgM class that bind either to cell surface-expressed or extracellular matrix antigens induce organ-specific damage in type II hypersensitivity diseases. More systemic autoimmune diseases are classified as type III hypersensitivity diseases and are characterized by circulating IgG and IgM as well as IgA that binds soluble antigen and forms immune complexes. Antibody-mediated autoimmune diseases include many different pathogenic mechanisms [reviewed in detail by Ref. (1)]. Among these are complement activation, recruitment of immune cells *via* Fc receptors (leading to antibody-dependent cellular cytotoxicity, opsonization, phagocytosis, and overall immune-mediated damage and inflammation), cross-linking of antigen that leads to immune complex formation or endocytosis of transmembrane antigen, or the direct modulation of antigen function. With the exception of the latter, these mechanisms rely on antibody class and subclass, particularly on the sequence and structure of the constant regions. Single amino acids in the constant region of IgG4 are responsible for structural differences that render it unable to exert most of the known pathogenic functions of antibodies. Despite their immunological inertness, several well-described autoimmune diseases are caused by IgG4 subclass autoantibodies, such as pemphigus, muscle-specific kinase (MuSK) myasthenia gravis (MG), and thrombotic thrombocytopenic purpura (TTP), and in recent years the number of potential IgG4 autoimmune diseases has rapidly grown. By now, 14 autoantigens are known that are targeted by IgG4 subclass autoantibodies; these antigens are found throughout the body (Table 1), with more than half being located in the central and peripheral nervous system. Whether all of the newly described IgG4 autoantibodies are pathogenic at all, if so by what mechanism, and if they are the sole pathogenic entity in their disease are essential questions that have been addressed to variable extent in the different fields. Here, we review what is known about IgG4 autoantibodies within the scope of IgG4 autoimmunity and attempt to analyze and classify proposed IgG4 autoimmune diseases based on a set of modified Witebsky postulates to validate IgG4 pathogenicity.

**Table 1:** Classification of IgG4 autoimmune diseases.

Disease	Antigen	Epitope	Other antibodies	Antibody binding to affected organ	Pathogenic mechanism of IgG4	Active immunization or passive transfer model	Biopsy/imaging finding	Other pathogenic mechanisms	References
**Class I diseases**									

MuSK-MG	MuSK	Ig-like domain 1,2, CARD domain	10% IgG1, 2	Neuromuscular junction	Yes. Block of MuSK–Lrp4 and reduced AChR clustering, block of MuSK–ColQ interaction	Yes, passive transfer of serum and IgG4, active immunization	Pre- and postsynaptic abnormalities at the NMJ	IgG1/2? MuSK endocytosis? Block of retrograde signaling?	(2–28)

CIDP	CNTN1	Ig-like domains (protein core, glycosylation independent)	IgG2, 3	Paranodal axoglial junctions of motoneurons	Yes. Block of contactin/Caspr and NF155 interaction, paranode dismantling	Yes, passive transfer of IgG4	Nerve: transverse band loss and paranodal loop detachment	N/A	(29–35)

Pemphigus foliaceus	Dsg1	N-terminal EC1 and EC2 domains, others	IgG1, 2, 3, IgA	Keratinocytes mostly in superficial layers of the skin	Yes. Block of cell adhesion, cell sheet dissociation in cultured human keratinocytes, and human skin explants. Pathogenicity was reduced after depletion of IgG4	Yes, several different models, e.g., passive transfer of cloned patient abs (IgG4). Also passive transfer from pregnant women to the fetus	Antibodies in circulation, patient skin and mucosal keratinocytes	IgG1, Dsg clustering and endocytosis, and keratinocyte signaling	(36–57)
Pemphigus vulgaris	Dsg3			Keratinocytes mostly in basal/parabasal layers of the skin					

Thrombotic thrombocytopenic purpura	ADAMTS13	5 small solvent-exposed loops in the spacer domain, others	IgG1, 2, 3, IgM, IgA	IgG in blood circulation (ADAMTS13 is a secreted protease)	Yes. Cloned IgG4 blocked ADAMTS13 protease activity which leads to von Willebrand Factor (vWF) accumulation and microthrombosis	Yes, transfection with recombinant anti-ADAMTS13 scFv cloned from patients, passive transfer of mAbs to baboons	IgG4 levels associated with relapse, circulating antigen/antibody complexes, some patients have exclusively IgG4	Yes. IgG1 cloned from patients also blocked ADAMTS13 activity. IgG, IgM: other mechanisms may include complement activation and clearing of ADAMTS13 from the circulation	(58–69)

**Class II diseases**									

Encephalitis, Morvan’s syndrome	Lgi1	Leucine-rich repeat and EPTP repeat domains	IgG1, 2	Synaptic cleft of CNS neurons, hippocampal neurons	Unclear. Serum: block of Lgi1–ADAM22 interaction and reduction of AMPA receptors *in vitro*, excitation of hippocampal CA3 pyramidal cells *ex vivo*	N/A	Brain atrophy in encephalitis-associated regions	CD8+ T cells, complement activation	(70–78)

CIDP, AIDP	Neurofascin 155	Fibronectin type III domains (FN3, FN4), N-glycosylated	IgG1,2, 3, IgM, and IgA	Central and peripheral paranodes	No. Suspected: block of NF155–CNTN1–CASPR1 interaction	N/A	Sural nerve: paranodal demyelination	N/A	(32, 79–85)

CNS and PNS disorders	CASPR2	N-Terminal discoidin and laminin γ1 modules of Caspr2 (glycosylation independent)	IgG1 in 12–63%	Juxtaparanodal region of myelinated axons, hippocampal GABAergic interneurons	No. Suspected: serum could affect Gephyrin clustering by blocking TAG1–Caspr2 interaction	N/A	IgG depositions in brain biopsy of 1 patient (but also complement)	Complement depositions and immune cell infiltrates in brain biopsy in 1 patient	(86–96)

Membranous nephropathy	PLA2R	Conformational epitope in tertiary structure of CysR domain, some also in CTLD1 and CTLD7	IgG1,3	Podocytes (kidney)	No. Suspected: atypical complement activation *via* the lectin pathway by IgG4 (serum/biopsies/purified IgG4, data not published). Block of podocyte adhesion to collagen IV?	No, due to technical challenges: PLA2R is not expressed in rodent podocytes	PLA2R-IgG in patient kidney biopsies	IgG1/3, classical complement activation	(97–109)

Membranous nephropathy	THSD7A	N/A, conformational epitope	IgG1,3	Podocytes (kidney)	Unclear, possibly a functional block of cell adhesion or altered signal transduction. Changes in architecture in cultured podocyte cells induced by IgG. Detachment and apoptosis of THSD7A overexpressing HEK293 cells	Yes, passive transfer of purified human or rabbit anti-THSD7A to mice, absence of complement deposition, altered podocyte architecture, increased stress fiber formation, activation of signaling at focal adhesions	Renal biopsy: IgG4 staining	IgG1/3? Complement staining (C5b-9)	(110–118)

**Class III diseases**									

Goodpasture syndrome	Type IV collagen	Alpha 3 chain of NCI domain	IgG1, 2, 3 (in few patients)	Glomerular basement membrane (kidney), lungs not tested	N/A	N/A	IgG deposition in glomerular basement membrane (kidney biopsy)	N/A	(119–123)

CIDP	CASPR1	N/A	IgG1	Paranodes (murine teased fibers)	N/A	N/A	Axonal degeneration		(35, 124)

DPPX encephalitis	DPPX	Extra- and intra-cellular domains (DPPX-L/S/X)	IgG1,2	Somatodendritic and perisynaptic neuronal surface, hippocampus, small intestine	No. Modulation/loss of DPPX and Kv4.2 by total IgG likely by IgG1/2. Hyperexcitation of enteric neurons.		Cerebrospinal (CSF) pleocytosis, increased IgG index or oligoclonal bands, and abnormal brain MRI	Modulation/loss of DPPX and Kv4.2 by total IgG likely by IgG1/2. Hyperexcitation of enteric neurons	(125–129)

Iglon5 parasomnia	Iglon5	Ig-like domain 2 (non-glycosylated)	IgG1, 2	Rat brain neuropil	N/A	N/A	Brainstem, hypothalamus: neuronal loss, deposits of hyperphosphorylated tau	IgG1 induces endocytosis of Iglon5	(130–135)

*ADAMTS13, disintegrin and metalloproteinase with thrombospondin motifs 13; Caspr, contactin-associated protein; CIDP, chronic inflammatory demyelinating polyradiculoneuropathy; CNTN1, contactin 1; DPPX, dipeptidyl peptidase-like protein 6; Dsg1, desmoglein 1; Dsg3, desmoglein 3; Ig, immunoglobulin; Iglon5, IgLON family member 5; Lgi1, leucine-rich, glioma-inactivated 1; Lrp4, low-density lipoprotein receptor-related protein 4; MUSK, muscle-specific kinase; PLA2R, phospholipase A2 receptor; scFv, single chain variable region fragments; THSD7A, thrombospondin type-1 domain-containing 7A; vWF, von Willebrand factor*.

## IgG4: From Structure to Function

### IgG4 Structure

IgG is the predominant antibody class and one of the most abundant glycoproteins in the human plasma with concentrations of approximately 7–15 g/L. In humans, four subclasses are known, IgG1, 2, 3 and 4, which are named in descending order of frequency (136). IgG4 is the least common IgG subclass, comprising only 5% of total IgG (137, 138). It is important to keep in mind that there is a different distribution in mice which are often used as animal models for pathogenicity, here the IgG1 subclass is the non-complement fixing subclass equivalent to IgG4, and in addition there are complement fixing IgG2a, IgG2b, and IgG3 subclasses (139–141). Also, there is different capability of human IgG to bind to rodent complement or Fc receptors, which needs to be considered in passive transfer models, e.g., by additional transfer of human complement (142).

The four human IgG subclasses share a similar structure with over 90% sequence homology. IgGs are heterotetramers, consisting of two heavy (50 kDa) and two light chains (25 kDa) of either κ or λ type. The heavy chain consists of three constant domains (C_H_) that mediate the antibody effector functions, a hinge region between C_H_1 and C_H_2, and one N-terminal variable domain (V_H_), which recognizes antigen. Similarly, the light chain is comprised of one variable (V_L_) antigen binding and one constant (C_L_) region. The V_H_ contains three complementarity-determining regions (CDR 1–3). These three CDRs, and particularly the CDR3, are hypervariable to allow for improved and highly specific binding of antigen. Increased mutation rates at this genetic locus indicate antigen-driven somatic mutations.

Disulfide bridges connect the light and heavy chains to form half-antibodies (HL), and two HL are joined together by covalent and non-covalent interactions between the heavy chains to form a whole antibody (H_2_L_2_). The heavy chains are mainly connected by disulfide bridges in the hinge region, which vary in numbers between the IgG subclasses. IgG1 and IgG4 have two disulfide bridges in the hinge region, while IgG2 has four and IgG3 11 (143–145). The disulfide bonds that connect heavy and light chains also vary between subclasses, in IgG1 they connect the n-term of the C_L_ domain with the c-term of the C_H_1 domain, while in IgG2–4 the c-term of the C_L_ domain is connected to the n-term of the C_H_1 region.

**Figure 1:**
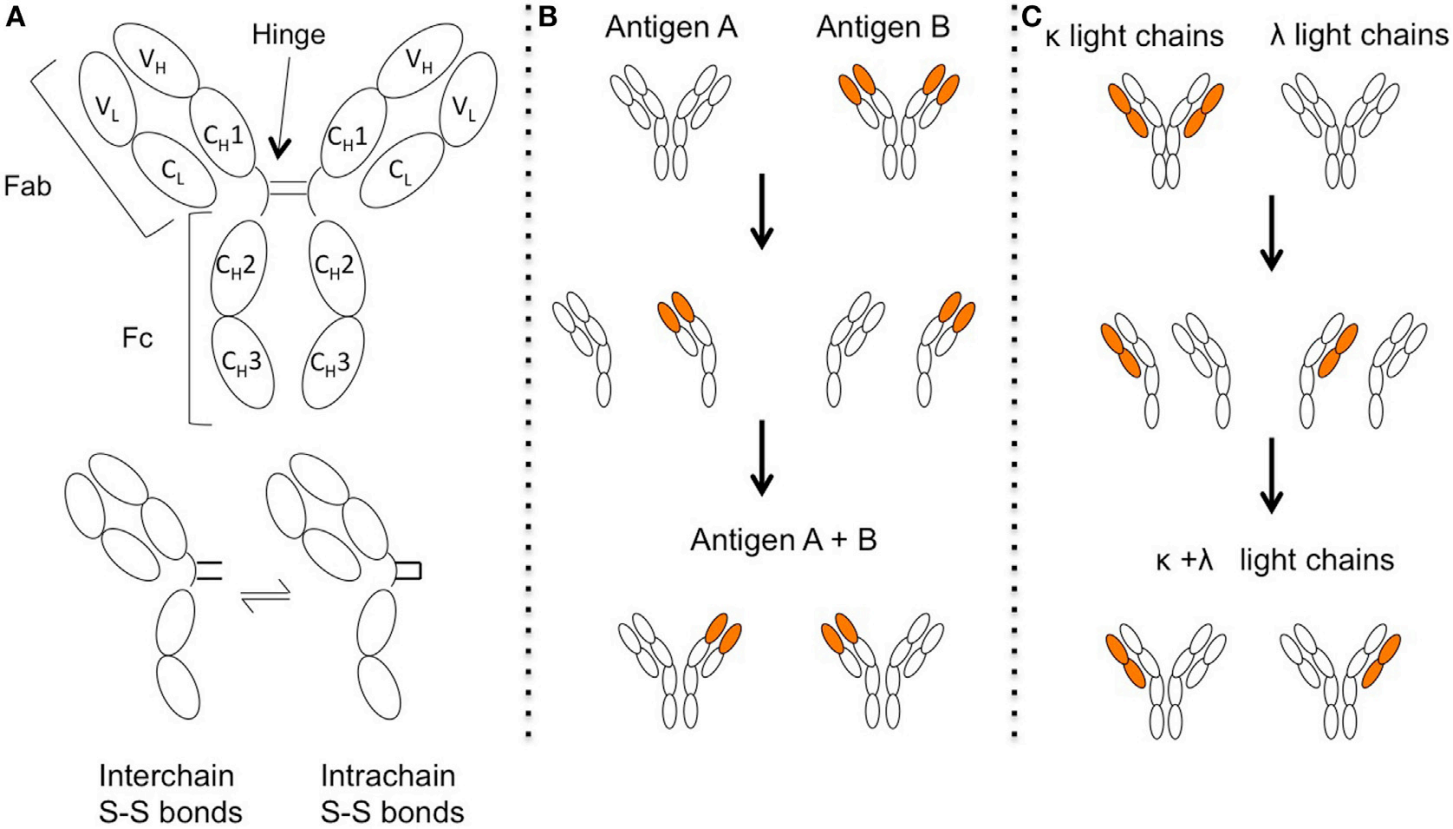
The structure of IgG4 allows for Fab-arm exchange (FAE). **(A)** Structure of IgG4. One single amino acid change in the IgG4 hinge allows for greater structural flexibility and two different hinge isomers, with either interchain disulfide bonds that connect the two different heavy chains, or intrachain disulfide bridges within the same heavy chains. Under reducing conditions, the two isoforms are in a dynamic equilibrium. Figure adapted from Ref. (147). **(B)** When the two half-antibodies (HL) are not connected by interchain disulfide bonds they can then split up. HL of different idiotypes recombine randomly, resulting in antibodies that recognize two different antigens and are bispecific. **(C)** Antibodies are produced with light chains of either κ or λ type. Upon FAE, antibodies with both κ and λ light chains can be generated.

Upon enzymatic digestion with papain, antibodies are divided into three fragments, one Fc fragment (“Fragment crystallized”), consisting of the C_H_2–C_H_3 of both heavy chains including the hinge, and two Fab fragments (“Fragment, antigen binding”), consisting of the C_H_1, V_H_, C_L_, and V_L_. The corresponding regions in the intact antibody are thus named “Fab” or “Fc” region [Figure 1A, reviewed by Ref. (146)]. Particularly interesting in IgG4 is the hinge and Fc region; while there is a high degree of homology between the IgG subclasses, there are single amino acid differences that have immediate consequences for IgG4 structure and effector function.

### Functional Characteristics of IgG4: Immunological Inertness and Fab-Arm Exchange (FAE)

Single amino acid changes in the C_H_2 region (exchange of a proline for a serine at position 331, “P331S”) prevent binding of the protein C1q (148, 149), the initial binding molecule of the classic complement cascade, and together with further changes (single amino acid replacements of L234F, P331S, and A327G) also cause a reduced binding to activating Fcγ receptors, but not to the inhibitory FcγRIIb (150–155). The exchange of a proline for a serine in the IgG4 hinge region at position 228 compared to IgG1 (P228S) leads to greater stereometric flexibility. This flexibility allows disulfide bridges in the hinge to additionally form within the same heavy chain (“intra-chain” disulfide bonds) instead connecting the two heavy chains (“inter-chain” disulfide bonds). Under reducing conditions, these states are dynamic and in equilibrium [Figure 1A (156–158)]. Together with reduced non-covalent interactions between the heavy chains in the C_H_3 region caused by an arginine instead of lysine (R409K) (159, 160), the two HL can dissociate (161–163) and recombine with other HL, in a process termed FAE [Figure 1B (161, 164–166)]. The resulting antibodies are bispecific and can cross-link two different antigens instead of two antigens of the same kind [Figure 1B (166, 167)]. As a consequence, IgG4 is not able to activate the classic complement pathway or activate immune cells as discussed in the part of the protective role of IgG4 in immunity. The following reviews are suggested for further reading on IgG4 structure (147, 168–170).

### FAE: Regulation and Kinetics

Fab-arm exchange seems to occur spontaneously in the body, since antibodies that are bispecific for two different allergens can be found in allergy patients (166). Here, endogenous IgG4 was able to cross-link two different allergens suggesting that an exchange between two distinct allergen-specific IgG4 molecules (birch and cat allergen) had taken place. FAE also occurred between human IgG and therapeutic, humanized antibodies (natalizumab) given as therapy to patients; here, the therapeutic antibodies exchanged half-molecules *in vivo* with endogenous IgG4 (171, 172). Bi-specific IgG4 was also detected in healthy probands, as IgG4 possessing both a λ and a κ light chain simultaneously was found in their serum [Figure 1C (173)]. FAE is inducible *in vitro* using reducing agents, such as glutathione (GSH) at concentrations of 1 mM and less (166, 171, 174–176). It is not known if and how FAE is regulated, or where it occurs in the body. The concentrations of GSH in human serum are too low to allow for efficient FAE, but other yet unidentified compartments in the body might provide the required GSH concentrations, e.g., human red blood cells, as they contain approximately 1 mM GSH (177).

*In vitro*-induced FAE occurs rapidly, in less than an hour (167), but *in vivo* it could take hours to days. The therapeutic monoclonal anti-integrin antibody natalizumab was found to exchange Fab arms in patients with endogenous IgG4 (172), with quantitative data obtained by injection of natalizumab into rats demonstrated an exchange half-life of less than 6 h (178). Human IgG4 has a metabolic half-life of around 21 days (179). Calculations led by Peter Molenaar based on these findings using first-order kinetics suggest that up to 99% of IgG4 should be bispecific in the body (17). There is further evidence: in IgG4 the κ light chain is more commonly used with a 3:1 κ: λ ratio. After FAE, IgG4 can harbor a κ and a λ light chain simultaneously (Figure 1C). The chance of a κ-half molecule to exchange with another κ-half-molecule is, therefore, more likely than the exchange with a λ-half-molecule, leading to a “silent” bispecificity hidden in the κ/κ and λ/λ fractions (173). The exchange is thought to be random. Calculating recombination probabilities using a Punnet square and assuming a 100% exchange rate, we expect a ratio of 9:6:1 (κ/κ:κ/λ:λ/λ). That means 37.5% of IgG4 would be κ/λ bispecific, which is close to the values measured in one study with healthy individuals (173). Taken together, it suggests that the majority of IgG4 in the human body is bispecific under normal circumstances. Despite the reduced avidity of IgG4, autoantibody binding can be demonstrated in tissue- and cell-based assays *in vitro*, e.g (3, 72, 88, 99)., perhaps due to the high affinity of the antibody with a Kd that can be in the picomolar range (180). Bispecificity also has functional consequences, as bispecific IgG4 is unable to divalently bind and cross-link one single species of antigen.

### IgG4 Fc–Fc Interactions

The Fc region of IgG4 is also able to directly interact with IgG by Fc–Fc interactions, but only if the binding partner is immobilized on a solid phase (181–183). The relevance of this interaction is not understood, but notably occurs in rheumatoid arthritis, IgG4-related disease (IgG4-RLD), and membranous nephropathy (MN), the latter being proposed as an IgG4 autoimmune disease (184–186). It is possible that IgG4 containing immune complexes could form by this mechanism; these would not otherwise form by divalent binding and cross-linking due to the bispecificity of the antibody (163). Modifications of the Fc region of IgG4 induced the formation of artificial hexameric complexes *via* non-covalent interactions that were able to bind C1q and activate the classical complement pathway (187). Whether this is relevant for pathophysiology of IgG4 is unclear.

### IgG4 Has a Protective Role in Immunity

**Figure 2:**
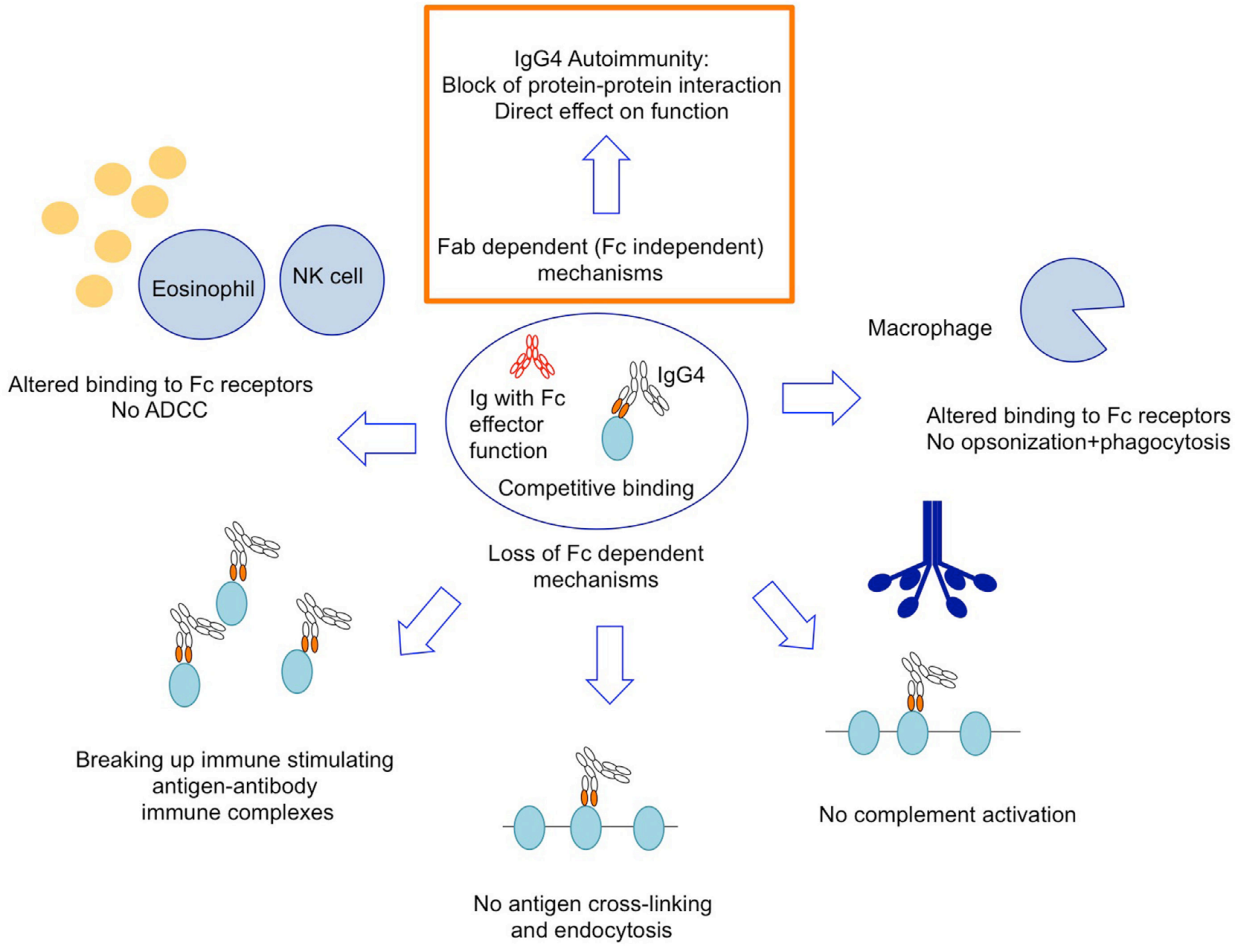
IgG4 autoantibodies rely on pathogenic mechanisms that are independent of Fc effector function or multivalent binding.

**Figure 3:**
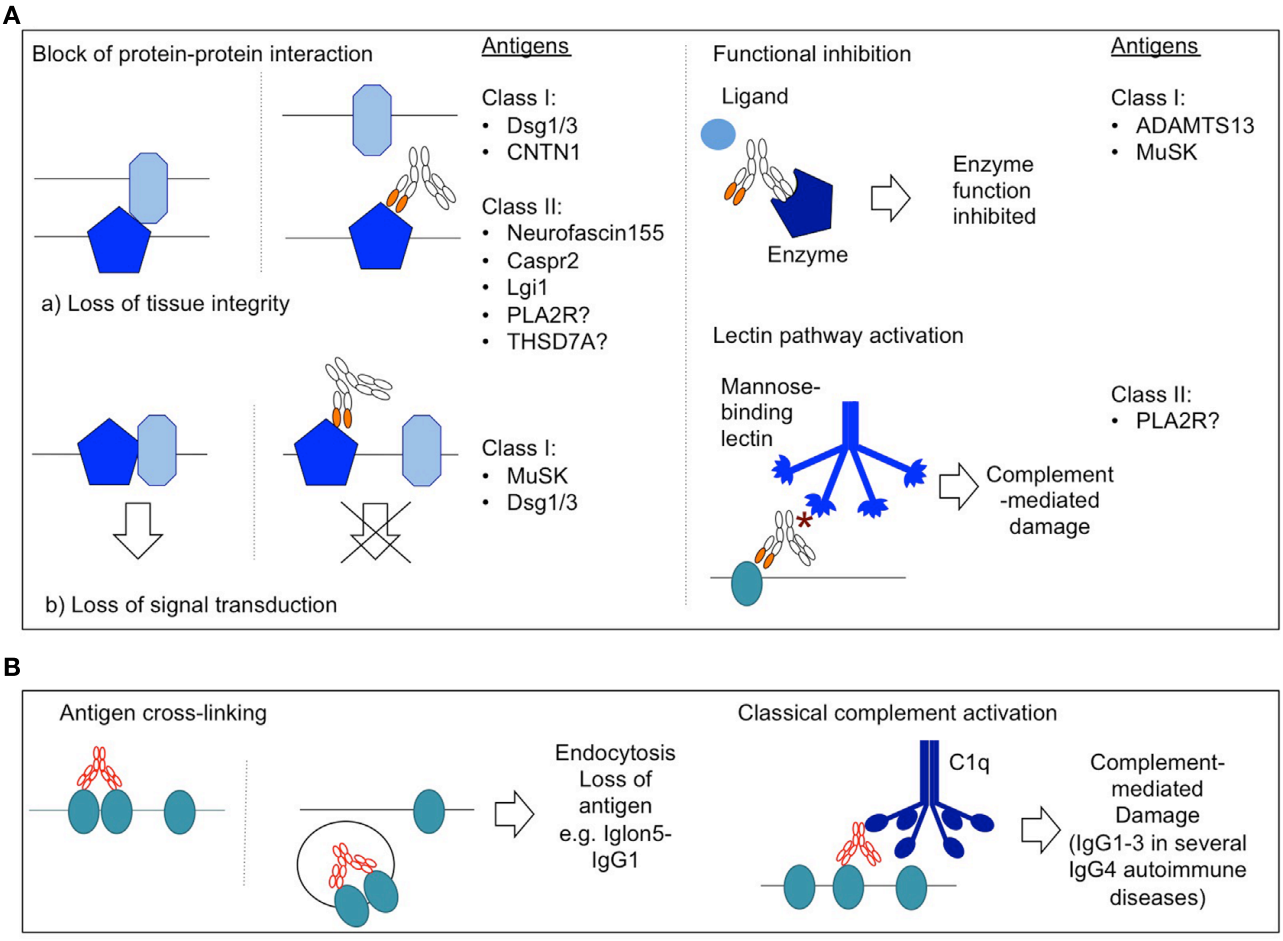
Selected pathogenic mechanisms of IgG autoantibodies. **(A)** Pathogenic mechanisms of IgG4 autoantibodies. * = hypogalactosylated glycan side chain of IgG4. **(B)** Selected pathogenic mechanisms of IgG1–3 autoantibodies.

IgG4 and IgE are usually produced in response to chronic exposure to antigens (188). The production of IgE and IgG4 is stimulated by T_h_2 cytokines IL-4 and IL-13. Furthermore, the additional presence of IL-10 is thought to tip the balance toward IgG4 production and IgE inhibition (189–192). In addition, secretion of IL-10 by regulatory T cells was also found to induce IgG4 rather than IgG1 production (193) and a majority of isolated IL-10-producing regulatory B cells produced IgG4 in contrast to other B-cell subsets (194). These findings link IgG4 with anti-inflammatory tolerance mechanisms [discussed in more detail here (147)]. At present, most literature reveals that IgG4 has an anti-inflammatory function. It has been shown that its levels only rise slowly after chronic exposure to antigen (195–197) or after allergy immunotherapy (198). Thereafter, IgG4 seems to protect against antibodies of other IgG subclasses by competition for antigen without exerting an effector function, thus blocking the epitope to prevent the harmful effect of other antibody classes or subclasses [Figure 2 (196–204)]. It has also been shown that passive transfer of IgG4 derived from hyperimmune beekeepers is clinically protective in allergy patients (205, 206) and protects mice from the effects of lethal africanized honey bee venom (207). Similarly, IgG4 subclass autoantibodies against the AChR protect against the pathogenic effects of IgG1 of the same idiotype in rhesus monkeys (166). The immunological inertness and the anti-inflammatory effect of IgG4 make it an unlikely subclass in the context of autoimmunity (Figure 2). In many cases [although there are exceptions (49)], antigen cross-linking and endocytosis in the context of autoimmunity rely on divalent binding [Figure 2 (208–213)], which IgG4 cannot do (2, 3, 166). IgG4 does not form immune complexes that would stimulate antigen-presenting cells and mount an immune response nor does it activate the classical complement system (166, 170, 214). Furthermore, IgG4 inhibits immune precipitation by IgG1 antibodies (163, 200). Only pathogenic mechanisms that are independent of Fc effector functions can be exerted by IgG4, e.g., a block of protein–protein interaction or a direct activation or inactivation of enzymes or receptors by competitive or allosteric binding (Figure 3A).

## Definition and Validation of IgG4 Autoimmune Diseases

Autoantibodies are in general considered pathogenic when they bind an extracellular antigen in the target organ, e.g., the extracellular portion of a transmembrane antigen, and have a direct pathogenic effect, either directly on the target antigen (e.g., by induction of endocytosis or blocking of antigen function) or on the whole organ by recruitment and activation of complement or immune cells that attack the tissue (1, 215). Antibodies against intracellular antigens are not considered to be pathogenic as they are generally inaccessible (215), although they can be valuable biomarkers (216). IgG4 subclass autoantibodies are certainly unexpected culprits in autoimmunity. Particularly, as IgG4 can arise during an immune-dampening response to pathogenic antibodies of other classes and subclasses, candidate diseases should be carefully investigated to determine the pathogenic status of autoreactive IgG4. Using a modification of the postulates by Witebsky, Rose and Bona, and taking into account remarks by Naparstek and Plotz (215, 217, 218), the individual IgG4 autoantibodies in IgG4 autoimmunity were tested to validate the status of IgG4 autoimmunity.

Several aspects should be considered to define IgG4 autoimmunity:

(A) Indicators for antibody-mediated autoimmunity
  1. Autoantibodies with specificity for an extracellular antigen are present in the affected organ.
  2. A pathogenic mechanism for antibodies is demonstrated *in vitro*.
  3. The autoimmune disease can be reproduced in experimental animals by passive transfer of patient serum or purified abs or active immunization with antigen.
  4. Clinical cues for autoimmunity are present such as HLA association, genetic clustering of other autoimmune diseases in the family, and the clinical improvement of the patients after therapies that reduce autoantibody levels, such as plasma exchange or B-cell depletion therapy.
(B) Specific indicators for IgG4 pathogenicity
  1. Autoantibodies of IgG4 subclass against extracellular antigen are present.
  2. A pathogenic mechanism for IgG4 can be demonstrated *in vitro*. IgG4 could be purified from patient serum, and the purity of isolated IgG4 should be validated; ideally also the IgG4-depleted IgG1–3 fraction should be used as a control group to ensure the effects derived from IgG4. Alternatively, cloned patient antibodies from IgG4+ B-cells could be used, although these may not be representative of the whole autoimmune B-cell population.
  3. Reproduction of disease in animals by passive transfer of purified IgG4 from patients, monoclonal IgG or single chain variable region fragments (scFv) cloned from IgG4+ B-cells of patients.

The 14 proposed IgG4 autoantibodies and their associated diseases were evaluated according to these aspects (Table 1). With particular focus on the pathogenicity of IgG4 autoantibodies, three classes emerged with different levels of evidence and also with different likeliness of IgG4 pathogenicity. *Class I diseases* fulfill two or three postulates of IgG4 pathogenicity (B.1–3), here the pathogenicity of IgG4 autoantibodies could be proven by passive transfer of patient IgG4, or an IgG4 pathogenic mechanism was demonstrated *in vitro* (in most cases this is was a blocking effect). *Class II diseases* fulfill one postulate for IgG4 pathogenicity (B.1), but there exists substantial circumstantial evidence that would be in line with pathogenicity of IgG4. Examples would be if IgG4 levels correlated with disease severity or the presence of a known pathogenic mechanism that relies on functional blocking. *Class III diseases* still fulfill one postulate of IgG4 pathogenicity (B.1) but have the least supportive evidence for IgG4 pathogenicity, either because the pathogenic mechanism relies on divalent binding or is unknown. At this point, a conclusion regarding IgG4 pathogenicity cannot be drawn for class II and III diseases, and more studies are required to validate pathogenicity of their IgG4 autoantibodies.

### Class I Diseases

One of the best-studied IgG4-mediated diseases is pemphigus. Here, autoantibodies bind to transmembrane proteins of keratinocytes, which leads to loss of cell–cell adhesion and blistering of the skin (acantholysis). The antigenic targets define different subforms of disease. In pemphigus foliaceus, the antigenic target is desmoglein 1 (Dsg1), which is expressed at high concentrations in the superficial layers of the epidermis; in pemphigus vulgaris, the mucosal-dominant type antibodies target desmoglein 3 (Dsg3) in basal and parabasal layers of the skin. Patients with pemphigus vulgaris of the mucocutaneous type have antibodies against both Dsg1 and Dsg3 (38, 39). Anti-Dsg autoantibodies are predominantly IgG4 (36, 37). They bind to extracellular epitopes and disrupt cell–cell adhesion, as seen *in vitro* where they induced cell sheet dissociation of cultured human keratinocytes (57, 219) and human skin explants (51). Additionally, the disease could be reproduced by passive transfer of monoclonal patient-derived antibodies that were of IgG4 subclass (42, 43). This means that pathogenicity of IgG4 could be demonstrated in the absence of patient antibodies of other IgG subclasses. Also, depletion of IgG4 reduced pathogenicity of patient serum in one study (220). Nevertheless, this does not mean that IgG4 is the exclusive pathogenic entity, and one study demonstrated that cloned patient IgG1 against Dsg3 was as pathogenic as IgG4 (221). The role of other IgG subclasses in IgG4 autoimmunity is discussed below in more detail. There are in fact several different disease models to investigate pathophysiology of pemphigus by passive or adoptive transfer [reviewed by Ref. (52)]. Maternal antibodies can also transfer to the fetus and cause neonatal pemphigus (54). Antibodies to Dsg1/3 may also affect signal transduction pathways that affect cytoskeleton rearrangement and modulate cell adhesion in keratinocytes, and several targets have been proposed (51, 222–228). Indeed, the MAPK signaling was suggested as potential therapeutic target in pemphigus (225), which can in part modulate the disruption of cell adhesion caused by pathogenic pemphigus autoantibodies (229).

Muscle-specific kinase MG is an autoimmune disease of the neuromuscular synapse hallmarked by fatigable muscle weakness. The autoantibodies target MuSK, a protein at the neuromuscular junction. Approximately 90% of the antibodies are of IgG4 subclass (3, 16), and the pathogenic mechanism of MuSK IgG4 is known and can be demonstrated *in vitro* with purified IgG4 (2, 3). Notably, the pathogenic effect was exclusive for IgG4 subclass antibodies. Passive transfer of purified IgG4 reproduced the disease in experimental animals (4), as did active immunization of complement-deficient mice (5). There is also evidence for a genetic predisposition in these patients (14, 15). Binding of antibodies to MuSK similarly affects its function as tyrosine kinase and organizer of neuromuscular junction development and maintenance. As the binding of the agrin coreceptor low-density lipoprotein receptor-related protein 4 (Lrp4) to MuSK is blocked by MuSK-IgG4 (2, 3), MuSK autophosphorylation in response to agrin is inhibited (2), which impairs its ability not only to induce clustering of the AChR (3, 27) but also to maintain the preexisting clusters (3). This leads to reduced densities of the AChR at the synapse and reduced efficiency of neuromuscular transmission, thus causing fatigable muscle weakness. A recent study demonstrated that inhibiting MuSK dephosphorylation counteracted the effects of MuSK antibodies *in vitro*, which would be an interesting new therapeutic strategy (230). Interestingly, the MuSK antibodies also modify the cross talk between motoneuron and muscle, as presynaptic abnormalities were observed in patients and passive transfer animal models, and led to a reduced quantal release of acetylcholine neurotransmitter into the synaptic cleft (5, 21, 22, 26, 231). The affected retrograde signaling pathway is not known, but as Lrp4 may be part of a retrograde signaling pathway (232, 233), it is very likely that the Lrp4–MuSK interaction plays a role, perhaps by anchoring Lrp4 at the synapse.

Disintegrin and metalloproteinase with thrombospondin motifs 13 (ADAMTS13) is a protease in the blood circulation that cleaves von Willebrand factor (vWF). In TTP, antibodies against the spacer domain of ADAMTS13 inhibit proteolysis of vWF (60, 234), which leads to accumulation of vWF, binding to platelets and causing microthrombosis. Since the inhibitory antibodies recognize the spacer domain that is also required for binding to vWF, the functional block could be due to blocked protein–protein interaction. IgG4 (but also IgG1) antibodies cloned from TTP patients by EBV immortalization demonstrated pathogenicity *in vitro* in assays measuring ADAMTS13 activity (69). Also, several patient-derived scFv were shown to be pathogenic when expressed in mice, albeit the original subclass of the patient antibodies is unknown (59). There is also additional circumstantial evidence that pathogenic antibodies of IgG4 subclass exist in TTP as IgG4 levels are associated with relapse and some patients have exclusively IgG4 autoantibodies (235).

Contactin 1 (CNTN1), together with contactin-associated protein 1 (Caspr1), is expressed on the axonal surface and together they bind to neurofascin 155 on the surface of oligodendroglia (236–239). They are cell adhesion molecules at the axoglial junction of myelin sheaths that are important for efficient nerve impulse propagation along myelinated axons. Antibodies against CNTN1 of IgG4 and IgG1 subclass are present in chronic inflammatory demyelinating polyradiculoneuropathy (CIDP) (29, 34, 35, 124) and were proven to be pathogenic. Autoantibodies recognize CNTN1 in the paranodal axoglial junctions of motoneurons and block interaction of neurofascin 155 with CNTN1 in the CNTN1/Caspr1 complex (30). This leads to transverse band loss and paranodal loop detachment in the peripheral nerves. Pathogenicity of IgG4 was proven by passive transfer of purified IgG4 to Lewis rats (31) with loss of paranodal clusters that contain CNTN1, Caspr1, and neurofascin 155 and impaired motor nerve conduction.

### Class II Diseases

Membranous nephropathy, a cause of proteinuria and nephrotic syndrome, is hallmarked by immune deposits containing IgG [predominantly IgG4 antibodies to the phospholipase A2 receptor (PLA2R) in primary MN], autoantigens such as PLA2R, and complement beneath the basal surface of podocytes in the kidney. PLA2R-IgG4 was proposed to have a unique mechanism of complement activation *via* the lectin pathway (98, 99, 103, 108, 109). This activation is hypothesized to derive from galactose-deficient side chains in PLA2R-IgG4 (97, 98) that might recruit mannose-binding lectin (MBL), which is also seen in rheumatoid arthritis (240) and IgA nephropathy (241). MBL binding leads to the activation of the lectin pathway and formation of the membrane-attack complex (103). Preliminary data by Beck and Salant from experiments with purified IgG4 from patients with primary MN showed that more of the IgG4 glycan chains lacked terminal galactose residues than did the IgG4 from control subjects and demonstrated increased binding of MBL to PLA2R-IgG4 (108). Additional evidence comes from a more recent study, where MBL deposition in glomeruli correlated with IgG4 positivity in biopsies from Japanese MN patients (109). A recent study by the group of Andreas Kistler demonstrated hypogalactosylated glycan side chains for IgG4, IgG induced complement-mediated injury to PLA2R-expressing podocytes *in vitro* and its prevention by MASP inhibitors, which adds further evidence to this unusual mechanism (poster presentation at the 2017 American Society of Nephrology meeting). However, both the Kistler and the Ma studies have not yet been published in peer-reviewed journals and need further investigation. IgG4-mediated activation of the lectin pathway may not be the only pathogenic mechanism, as one patient with characteristic PLA2R-IgG4-positive MN was deficient for MBL but revealed complement activation *via* the alternative pathway (242), and a small fraction of patients is negative for PLA2R-IgG4 (243). Additionally, a monoclonal IgG3 kappa with specificity for PLA2R was able to cause MN in the native kidney and the allograft *via* the classical pathway (244). An additional blocking mechanism was proposed since antibodies against PLA2R in MN were found to block adhesion of podocytes to collagen IV (245). In another study, PLA2R, however, was not found to bind collagen IV or I in general (106, 246). Furthermore, attempts to reproduce the disease in rodents by passive transfer have been unsuccessful, since the podocytes of rodent glomeruli do not express PLA2R [summarized by Ref. (247)]. Therefore, further studies are required to validate this finding and pinpoint the mechanism to IgG4.

Thrombospondin type-1 domain-containing 7A is expressed in glomeruli in the kidney and is another antibody targeted in MN (110, 113, 114, 118). Histologically, a thickening of the glomerular basement membrane with IgG and complement deposition can be observed. Anti-THSD7A antibodies were predominantly IgG4 (but also IgG1) (110, 116) and *in vitro* were shown to affect cytoskeletal architecture with the formation of stress fibers in cultured primary murine podocyte cells and could also induce detachment of THSD7A overexpressing HEK293 cells (111). Passive transfer of patient serum to mice led to proteinuria with late complement activation, but transient proteinuria in the absence of complement involvement was induced after passive transfer of THSD7A affinity-purified patient antibodies (111). Passive transfer of anti-THSD7A antibodies generated in rabbits could reproduce disease independent of complement activation (112). Taken together, there is evidence that THSD7A antibodies affect podocyte attachment, perhaps by a (reversible) blocking effect, but also that complement activation *via* C1q or lectin-binding pathway as a second pathogenic mechanism plays a role.

In three different neurological disorders with IgG4 subclass antibodies, targeting leucine-rich, glioma-inactivated 1 (Lgi1), contactin-associated protein 2 (Caspr2), and Neurofascin155, respectively, a pathogenic mechanism has been suggested that is based on the block of protein–protein interaction (70, 80, 87, 93, 248). The predominant mechanism of IgG4 antibodies in class 1 IgG4 autoimmune diseases was found to be a blocking mechanism, which may also be associated with a subclass switch to IgG4. It should be kept in mind that a blocking mechanism is not *per se* restricted to the IgG4 subclass [e.g., Ref. (69)], although it is more commonly associated with IgG4 autoimmunity. In detail, Lgi1 is expressed at the synaptic cleft of CNS neurons and in hippocampal neurons in culture, like CASPR2, it is part of the voltage-gated potassium channel complex, and antibodies against Lgi1 are associated with limbic encephalitis (72, 77, 93, 249). Here, serum from patients with Lgi1 encephalitis led to a block of Lgi1–ADAM22 interaction *in vitro*, which caused reduced AMPA receptors and excitation of hippocampal CA3 pyramidal cells *ex vivo* (70). These effects were not yet proven to be IgG4 specific. Caspr2 is expressed in the peripheral and central nervous system, particularly in the juxtaparanodal region of myelinated axons and in hippocampal GABAergic interneurons, and autoantibodies are associated with different clinical syndromes (88, 91, 250–252). It is suspected that the antibodies block the interaction between Caspr2 and TAG-1 and affect inhibitory interneuron function, thus leading to hyperexcitability (87). Importantly, many patients harbor not only IgG4 subclass antibodies against Caspr2 or Lgi1 but also IgG1 and IgG2 (88). Antibodies to neurofascin 155 are thought to have a similar effect to antibodies against CNTN1, with a block of CNTN1/Caspr1–neurofascin 155 interaction affecting paranodal structure and causing peripheral neuropathies (30, 79, 81, 84).

### Class III Diseases

IgLON family member 5 (Iglon5) is a CNS antigen and is targeted by autoantibodies in Iglon5 parasomnia, which is a severe autoimmune disease characterized by abnormal sleep behavior. Here, a population of pathogenic IgG1autoantibodies induced endocytosis of Iglon5 (134) (discussed in more detail below). It was demonstrated that purified Iglon5-IgG4 as well as digested Fab fragments did not induce this mechanism. It cannot be excluded that Iglon5-IgG4 antibodies have an additional pathogenic mechanism, but at this moment there is no indication that they are pathogenic at all.

Similarly, in the case of dipeptidyl peptidase-like protein 6 (DPPX) antibodies, the described pathogenic mechanism is the loss of DPPX and associated Kv4.2 from the cell surface, perhaps by antigenic modulation, which would require divalent binding and cross-linking of antigen. Unless an additional pathogenic mechanism is found for DPPX-IgG4, or alternatively, it is demonstrated that the loss of DPPX and Kv4.2 results from a mechanism that does not rely on divalent binding, it is not likely that DPPX encephalitis is an IgG4 autoimmune disease.

Only one patient has been described with IgG4 subclass antibodies against Caspr1 [a second one if antibodies against complexed CNTN1/Caspr1 are considered (35, 124)], and at this point not enough data are available to estimate pathogenicity of IgG4. There are also insufficient data available regarding pathogenicity of IgG4 subclass anti-collagen type IV (alpha3NC1 domain) antibodies in Goodpasture syndrome, the most convincing circumstantial evidence being temporal occurrence and reoccurrence of IgG4 autoantibodies and lung hemorrhage (119).

In summary, the pathogenicity of IgG4 in most IgG4 autoimmune diseases has not been proven yet. A key experiment could be the isolation of IgG4 subclass antibodies from patient serum or plasmapheresis material, followed by passive transfer to a suitable animal model, or to use purified IgG4 in already established *in vitro* models of pathogenicity.

## Properties of IgG4 and IgG4 Autoimmune Diseases

One key pathogenic mechanism in antibody-mediated autoimmunity, especially in the peripheral and central nervous system, is the divalent binding, cross-linking, and endocytosis of antigen, as seen with antibodies against AChR, GlyR, NMDAR, and Iglon5 [Figure 3B (134, 208–210, 253)]. The effect is usually lost when using Fab fragments instead of whole IgG1, which underlines the relevance for multivalent binding. A possibility how IgG4 autoantibodies could remain pathogenic by cross-linking and endocytosis would be the loss of FAE, as then monospecific IgG4 could still cross-link and internalize antigen. An example could be the presence of point mutations or sequence variants in the IgG4 hinge or the C_H_3 region that would remove its ability to undergo FAE. This possibility was investigated in several IgG4-associated diseases. In IgG4-RLD, sequencing showed that the FAE abolishing variant K409 was not present (254), and a recent study demonstrated high levels of bispecific IgG4 in IgG4-RLD (255). Similarly, IgG4 autoantibodies in rheumatoid arthritis were found to be bispecific (256), and recently we were able to demonstrate the same for an example of IgG4 autoimmunity, as a large proportion of patient-derived MuSK autoantibodies were bispecific as well (17). These are only a few publications yet, and more studies are needed to validate the findings in additional IgG4-associated diseases. Until then, there is no indication that impaired FAE affects IgG4 in IgG4-associated disorders, which suggests that pathogenic mechanisms of IgG4 autoantibodies do not rely on divalent binding. Notably, the authors of the IgG4-RLD study suggest increased levels of FAE as a new biomarker. As IgG4-RLD is also associated with elevated levels of IgG4, and up to 99% of IgG4 are probably bispecific, it would be interesting to test a correlation between IgG4 concentrations and κ/λ bispecific IgG4. Enrichment of κ/λ IgG within the total IgG4 population (compared to κ/κ and λ/λ antibodies) could indicate a change in light chain usage.

### Blocking as a Main Pathogenic Mechanism of IgG4

Blocking mechanisms are independent of the Fc domain and as such are the main effector mechanisms for IgG4 autoantibodies (Figure 3A). The binding of antibody to antigen can cause the inappropriate activation or blocking of enzymes or receptors. As examples, blocking antibodies of IgG1–3 subclass prevent binding of the neurotransmitter acetylcholine to the acetylcholine receptor in AChR MG (257–259), blocking antibodies against thyroxin peroxidase reduce production of thyroid-stimulating hormone (TSH) thus causing hypothyroidism (260), while stimulating antibodies against the TSH receptor are associated with hyperthyroidism (261). Functional blocking as a pathogenic mechanism is found in not only the IgG4 autoimmune diseases TTP and MuSK MG but also pemphigus (2, 52, 60, 234).

Block of protein–protein interaction is overall considered a main pathogenic mechanism for IgG4 and was demonstrated in several class I and II IgG4 autoimmune diseases (Figure 3A). The consequence of such a block could be the block of a signal transduction pathway (MuSK, Dsg1/3) or the loss of tissue integrity (e.g., Dsg1/3, Caspr1, and CNTN1). Most IgG4 autoantibodies were found to block protein–protein interaction.

### Complement Activation

Another key mechanism of IgG1–3 autoantibodies is the recruitment of C1q protein and activation of the classical complement pathway, which leads to complement mediated damage, e.g., by antibodies against AChR or aquaporin 4 [Figure 3B (262–265)]. IgG4 does not bind C1q, but intriguingly, complement activation by the lectin pathway may emerge as a novel mechanism of IgG4 autoantibodies, as it is found in PLA2R-IgG4 with hypogalactosylated glycan side chains [Figure 3A (98, 99, 103, 108, 109)].

### IgG4 Autoimmune Diseases

IgG4 pathogenic mechanisms translate to a range of different disorders that are clinically very diverse. An excellent introduction to the different IgG4 autoimmune diseases can be found here (266). A selection of disease specific reviews is found here (9, 52, 80, 106, 235, 267–271). A good response to B-cell depletion therapy and plasmapheresis has been reported in several IgG4 autoimmune diseases (6–13, 32, 78, 272–274).

### Contribution of Other Antibody Classes and Subclasses

As IgG4 levels usually increase after repeated or strong exposure to antigen, a high level of IgG4 may coexist with (smaller quantities of) pathogenic antibody of other class or subclass (e.g., IgE or IgG1) and could have clinical implications for treatment decisions. Specifically, after the evaluation and classification of the proposed IgG4 autoimmune diseases, it emerged that in most cases several different antibody classes and/or subclasses are present in patient serum (Table 1), with varying degree of potential pathogenicity. Of note, IgG4 concentrations could potentially be overestimated depending on the choice of secondary antibody (16), and cross-reactivity between secondary antibodies against IgG4 and IgG2 is also known to occur. A correlation between overall antibody titer and antigen-specific IgG4 titer would increase confidence in the quantification data (3).

Furthermore, pathogenic mechanisms were described for serum or IgG from patients with IgG4 autoimmunity that are likely not caused by IgG4 subclass antibodies as they depend on divalent binding or Fc effector function (Figure 3B, endocytosis of antigen and complement activation) or have been demonstrated using purified or cloned patient IgG1–3 [e.g., Dsg3-IgG1 (221), ADAMTS13-IgG1 (69), Lgi1-IgG3 (275), MuSK-IgG1–3 (3), or Iglon5-IgG1 (134)]. Indications for complement activation were found in several disorders (Table 1).

Particularly interesting are Iglon5 autoantibodies in which an irreversible internalization of Iglon5 was shown. Here, a clear pathogenic mechanism of IgG1, depending on divalent binding (134), was shown. Iglon family members are glucosylphosphatidylinositol-anchored proteins that have a role in membrane stabilization. Iglon5 parasomnia also has an unusual association with tauopathy (130, 276, 277) linking autoimmunity to neurodegenerative diseases. Combined, these findings led to the current hypothesis that Iglon5-IgG1 antibodies, by antigenic modulation of Iglon5, cause a destabilization of the cytoskeleton. This may affect microtubule stability and hyperphosphorylation of associated tau protein that could lead to the observed tauopathy (276). Whether IgG4 subclass antibodies to Iglon5 are pathogenic at all is unclear, thus making it a class III IgG4 autoimmune disease.

Similarly, DPPX antibodies cause a reversible loss of cell surface-expressed DPPX and Kv4.2, thus leading to hyperexcitation of neurons (127). This requires divalent binding and cross-linking of antigens. As IgG4 is mostly bispecific [also in the context of IgG4 autoimmune diseases (17)], it is likely that DPPX-IgG4 is not able to divalently bind and cross-link DPPX. While the precise mechanism for downregulation of cell-surface-expressed antigen has not yet been demonstrated, it is likely that this is a function of IgG1–3 that causes internalization by divalent binding and cross-linking of antigen. This possibility could be explored by a modification of the experiment using purified IgG subclasses and Fab fragments.

Notably, 10% of MuSK antibodies are of IgG1 and IgG2 (and very little IgG3) subclass and were pathogenic *in vitro* (3). MuSK IgG1–3 pathogenicity *in vivo* could not yet be demonstrated, but can also not yet be excluded, as passive transfer experiments were impaired by the low concentration of MuSK antibodies in the IgG1–3 fraction that were below detection limit in the experimental animals (4). The exact mechanism of pathogenicity for MuSK IgG1–3 is unknown. MuSK-IgG1–3 were able to affect MuSK function in a cellular model of the muscle and neuromuscular synapse, the C2C12 mouse myotubes. Agrin stimulation leads to MuSK activation and clustering of AChRs, which can be quantified. MuSK antibodies impaired this mechanism and led to reduced clustering of AChRs, and while it is thought that MuSK-IgG4 induce this phenomenon by blocking MuSK–Lrp4 interaction (2, 3), it is unclear how MuSK IgG1–3 affected the AChR clustering as they do not interfere with Lrp4–MuSK interaction. Interestingly, MuSK antibodies (both IgG4 and IgG1–3) also disrupted preexisting AChR clusters that were induced by overexpression of Dok7 in C2C12 myotubes [(3) Koneczny/Vincent et al. unpublished data] independent of agrin signaling, which was also found using autoantibodies against Lrp4 derived from an active immunization mouse model (278). An earlier study suggested that Lrp4 is required to make MuSK susceptible to Dok7-induced activation and induction of AChR clustering, presumably with a role for AChR prepatterning before innervation (279). MuSK IgG1–3 could also have an effect on Lrp4, though it is not clear how, or MuSK antibodies may affect Dok7-mediated MuSK activation, perhaps by altering MuSK conformation, MuSK kinase activity and/or preventing binding of Dok7 to MuSK, as observed in CMS with a MuSK mutation (280). It would be interesting to study whether MuSK-IgG1–3 antibodies affect MuSK–Dok7 interaction.

Another potential mechanism of MuSK IgG1–3 is the induction of MuSK endocytosis, which is also suggested to be part of the normal response of MuSK to agrin stimulation (281–283). One study demonstrated that serum from MuSK patients led to the internalization of MuSK in mouse muscle cells using time-lapse microscopy and confocal microscopy (28). Two different studies could not reproduce these findings using different approaches (2, 3). However, different patients were tested in these studies, and only a few patients were investigated for endocytosis in all three studies, which is important, as there could be variability in the pathogenic mechanisms in the overall MuSK MG population. In addition, there were technical limitations in the later studies [including my own, as discussed there (3)], hence it is still very possible that MuSK IgG1–3 induce MuSK endocytosis. Further studies are indicated using purified MuSK IgG1–3, MuSK expressed in muscle cells (as transient expression in non-muscle cell environment could affect MuSK endocytosis), and perhaps more appropriate methodology.

In TTP, antibodies of IgG, IgM, and IgA class are present; these may convey pathogenicity by the accelerated clearance of ADAMTS13 from the circulation (234, 284). The functional block of ADAMTS13 activity was not restricted to the IgG4 subclass and could also be induced by IgG1 (69). Therefore, the pathogenic mechanism is not linked to the antibody subclass, but rather the epitope, as only antibodies that recognize the spacer domain are associated with an inhibitory role (234).

In patients with MN with anti-PLA2R abs, a subclass switch from IgG1 to IgG4 was reported over the course of the disease, with an inverse correlation with C1q involvement (285). It is thus clear that in the initial stage of the disease, IgG1 contributes to pathogenicity by activation of the classical complement pathway (100), which may then at later stages of the disease be replenished or replaced by IgG4 that may be protective, inert, or pathogenic (99).

In pemphigus, IgG1 antibodies against Dsg1 or 3 are present and they may also contribute to the blocking mechanism (221). Additional pathogenic mechanisms in pemphigus were proposed as desmosome disassembly by clustering and endocytosis of Dsg and stimulation of signaling pathways that modulate keratinocyte cell adhesion (50, 51). It is thought that multivalent polyclonal Dsg antibodies can cross-link and endocytose Dsg, which leads to Dsg depletion from desmosomes and failed cell adhesion (44–48). However, also monovalent pathogenic anti-Dsg3 antibodies led to a depletion of Dsg3, which suggests that this could also be a mechanism of potentially bispecific (and thus monovalent) Dsg3-IgG4 (49).

Taken together, the presence of more than one pathogenic entity in IgG4 autoimmune diseases is quite possible and makes sense, if we consider that a rise of antigen-specific IgG4 in theory could have been an attempt of the immune system to dampen an inappropriate answer to the antigen by a different antibody species. This could also have clinical consequences, as monitoring of different Ig class/IgG subclass-specific autoantibodies may provide a deeper understanding of the autoimmune response, the individual antibody levels or a ratio thereof could be useful as biomarkers for disease progression or the selection of appropriate therapy.

### Presence of Multiple Antibodies in Individual Cases

Two case reports describe the co-occurrence of MuSK MG or TTP with IgG4-RLD (286, 287), which brought up the interesting question whether these diseases might be related. The term IgG4 autoimmunity is relatively new and was introduced in 2015 (266). IgG4-RLD [which is also a relatively new term (288)] is at this point considered as a separate clinical entity. IgG4-related disease is not discussed extensively here, an excellent review is suggested for further reading (289) and also a review with focus on IgG4 in IgG4-related disease (290). Due to the rareness of the IgG4 subclass and its involvement in pathology, the possibility exists that IgG4-RLD and IgG4 autoimmunity could be part of the same spectrum. Both diseases are associated with IgG4, and both have a favorable clinical response to B-cell depletion therapy with rituximab (6, 32, 78, 272–274, 289). However, IgG4-RLD is defined by the formation of tumefactive lesions in target organs, which is not normally described in IgG4 autoimmunity, and by IgG4+ plasma cell infiltrates and increased serum IgG4 levels. Normal IgG4 concentrations are variable, but are thought to comprise 5% of total IgG (137, 138). As mean IgG concentrations vary between 7 and 15 g/L with a mean around 10 g/L, IgG4 concentrations are expected to be around 0.5 g/L (but can rise up to 100-fold in immune responses). IgG4 concentrations above 1.35 g/L are considered as a diagnostic marker for IgG4-RLD (291). In IgG4 autoimmunity, few studies have looked at serum IgG4 levels, but in one study total serum IgG4 was found to be elevated in a small fraction of pemphigus patients (220), which is notable, but only occurred in a minority of patients: three of 27 pemphigus vulgaris patients and three of 16 pemphigus foliaceus patients had IgG4 concentrations above the threshold of 1.35 g/L (or 135 mg/dL). In addition, the total IgG concentrations were reduced in these patients, likely a consequence of immunosuppressive treatment [pemphigus is routinely treated with immunosuppression (292)]. Perhaps the immunosuppressive treatment in combination with an on-going IgG4 autoimmune disease also increased relative IgG4 levels, which would be interesting to study in any IgG4 autoimmune disease using patient serum before and after treatment.

A key characteristic in IgG4 autoimmunity is the presence of antigen-specific autoantibodies of IgG4 subclass with direct pathogenic function. In IgG4-RLD, the role of IgG4 is not well understood, but it is thought that IgG4 may have a blocking, anti-inflammatory function. There is indirect evidence for an antigen-driven pathogenicity, namely the presence of oligoclonal bands in the CSF of patients and the presence of oligoclonal expansions of somatically hypermutated IgG4+ B-cell clones, but these were not associated with any known autoantigen (293–296). Only a few antigen-specific autoantibodies have been described that are also not consistently found in the disease, and the pathogenicity of these autoantibodies is not known (297, 298). Experiments with purified antibodies from patients with a pancreatic form of IgG4-RLD showed that IgG4 blocked pathogenic effects of IgG1 in a passive transfer animal model (299), and a recent study identified Annexin A11 as an antigenic target in some patients with autoimmune pancreatitis. The study findings also suggest that the pathogenic entity may be IgG1 and that IgG4 may be upregulated as an anti-inflammatory measure that blocks pathogenic IgG1 (300). More studies are needed to validate this finding, also for other subtypes of IgG4-RLD. However, the relatively clear pathogenic role of IgG4 in IgG4 autoimmunity and the potentially protective role of IgG4 in IgG4-RLD make it as a consequence not very likely that these disorders are (closely) related.

## Etiology of IgG4 Autoimmunity

The etiology of most autoimmune diseases and thus of most IgG4-mediated autoimmune diseases is not known, but there are a few interesting observations that could give a clue to potential factors and mechanisms that may contribute to immunopathogenesis.

### Environmental Antigens

In Europe, MuSK MG frequency correlates with geographical latitude (301), with few cases in northern countries but higher prevalence in the south. This pattern is shared by another IgG4 autoimmune disease, pemphigus (302, 303). The distribution might be coincidental, as epidemiology data of MG in the rest of the northern hemisphere do not reflect the same pattern. Or it could indicate an environmental and/or genetic factor, for example, different availability of an environmental antigen, or different climates with different exposure levels to parasites/helminths in the past could have influenced the type 1 hypersensitivity immune answer including the intensity of the IgG4 response. However, the most striking example for environmental antigen-induced autoimmunity comes from fogo selvagem, which is an endemic form of pemphigus that is found mainly in rural areas of Brazil, but also in Colombia and Tunisia (303, 304). Here, an association between insect bites and autoimmunity has been found. The bite of the sand fly (*Lutzomyia longipalpis*) exposes the patient to salivary proteins, specifically LJM11, against which most humans then develop antibodies. While LJM11 itself is a non-pathogenic environmental antigen, mounting an immune response against it is thought to be protective against parasites (specifically *Leishmania major*) that are also transmitted by the bite of the sand fly (305). Unfortunately, the responding antibodies against the non-pathogenic environmental antigen can also cross-react with self-antigen desmoglein 1 (Dsg1) in individuals with genetic predisposition (306, 307). The initial subclinical antibody response is thought to contain IgE, IgG1, and IgM (308–310), then a switch to IgG4 subclass occurs, probably associated with an intramolecular epitope spreading event (311) that is associated with disease onset (312, 313). Etiology of autoimmune diseases is rarely as well documented as in the fascinating case of fogo selvagem. It would be interesting to study if a similar mechanism could be at hand in other IgG4-mediated diseases. A test for antigen-specific IgE in IgG4-associated diseases could give first indications if this was the case. Indeed, a potential link to allergy recently emerged for a different IgG4-associated disease, IgG4-RLD. An association of IgG4-RLD with a history of allergy was already suspected (314), then a polyclonal response to multiple non-infectious environmental antigens was discovered (315) and a rise in IgE and eosinophil levels was found to predict relapse and to have potential use as diagnostic and prognostic biomarkers (316).

### Infection

Several different (potential) IgG4 autoimmune diseases may have a link to infection, generally thought to be conveyed by molecular mimicry, where antibodies against pathogens cross-react against self-antigen. The autoreactive B-cells in pemphigus showed a shared VH1–46 gene usage, and these were associated with few somatic mutations, which suggests that naïve B-cells that use VH1–46 genes are prone to react against Dsg3 (55). VH1–46 B cells are also associated with an increased reaction toward rotavirus capsid protein VP6 and may thus confer a protection toward infection, highlighting the potential for rare cross-reactive clones to trigger the onset of pemphigus autoimmunity (317, 318). In one of two known CIDP patient with Caspr1-IgG4, disease onset was 10 days after preceding virus infection (common cold) (124). Patients with DPPX encephalitis usually experience severe prodromal symptoms of diarrhea and weight loss (median 20 kg), sometimes accompanied by headache or mood disorder, then within a few months the patients develop a range of neurological symptoms (125, 127, 128). DPPX is strongly expressed in not only the myenteric plexus, a mesh of neurons in the gastrointestinal tract, but also the hippocampus, cerebellum, and striatum. The curious shift in symptoms is reminiscent of a gastrointestinal infection that leads to the mounting of an autoimmune attack against the myenteric plexus *via* molecular mimicry or bystander attack mechanisms, as seen, e.g., in *Campylobacter jejuni* infection that causes the Guillain–Barré syndrome [reviewed, e.g., by Ref. (319)]. So far, no pathogen was found to be associated with the prodromal symptoms in DPPX-encephalitis, and it is also possible that this shift may have different causality, but it is an intriguing possibility to keep in mind.

In case of PLA2R antibody-positive MN, infection and molecular mimicry were proposed to potentially contribute to immunopathogenesis, as there is partial homology between PLA2R peptides and bacterial cell wall enzymes of Clostridium species (101, 320, 321). A link between infection and Goodpasture’s disease with antibodies against non-collagenous domain 1 of α3 chain of type IV collagen was also described (322, 323). One case of TTP was associated with Epstein–Barr virus reactivation (324), another case disease onset was associated with an influenza A infection (325) and another with dengue virus infection (326).

### Vaccination

There are a few cases of TTP onset or relapse after vaccination (327–329). This is interesting, as a related disease, immune thrombocytopenic purpura, which has antibodies against platelets, is also associated with vaccination (330).

### Malignancy

Overexpression of antigen by tumors can trigger an autoimmune response. THSD7A-positive MN is associated with malignancy, as THSD7A itself may also play a role in certain cancers (331) and specifically two cases were described, one with gallbladder carcinoma, one with endometrial cancer, where THSD7A was overexpressed in the tumor and taken up by follicular dendritic cells in a regional lymph node (106, 113, 115, 116).

### Summary and Conclusion

In recent years, many IgG4 autoantibodies were discovered, particularly against neuronal targets. By now, 14 different antigen targets have been described and suggested to play a causative role in “IgG4 autoimmunity” (266). Using a modified version of the Witebsky postulates to identify IgG4 autoimmune diseases with proven pathogenicity of IgG4 autoantibodies, the diseases were classified into three categories: class I with proven pathogenicity of IgG4 autoantibodies (including TTP with ADAMTS13 antibodies, pemphigus vulgaris with anti-Dsg3 antibodies, pemphigus foliaceus with anti-Dsg1 antibodies, MuSK MG and CIDP with anti-CNTN1 antibodies), class II where pathogenicity of IgG4 is highly suspected (anti-Lgi1- and Caspr2-associated encephalitis/Morvan’s syndrome, CIDP with anti-neurofascin155 antibodies, and MN with anti-PLA2R or anti-THSD7A antibodies), and finally class III with diseases where IgG4 pathogenicity has not been studied extensively yet (anti-Caspr1-associated CIDP and Goodpasture syndrome with IgG4 antibodies against Collagen IV alpha3NCI) or that have a main pathogenic mechanism that relies on divalent binding, cross-linking and endocytosis of antigen (Iglon5 parasomnia and anti-DPPX encephalitis). Class II and III diseases require further investigation to determine the pathogenicity of IgG4, ideally by passive transfer experiments with purified patient IgG4. Evaluation of the diseases suggests that IgG4 is mainly pathogenic by blocking protein–protein interaction, but that in many cases non-IgG4 autoantibodies are present that may contribute to pathogenicity by other mechanisms, in many cases activation of classical complement is suspected. This may have clinical consequences for treatment decisions. The possibility of an association between IgG4 autoimmunity and IgG4-RLD was discussed but found unlikely.

## Author Contributions

The author confirms being the sole contributor of this work and approved it for publication.

## Conflict of Interest Statement

The author declares that the research was conducted in the absence of any commercial or financial relationships that could be construed as a potential conflict of interest.

## Abbreviations

ADAMTS13, disintegrin and metalloproteinase with thrombospondin motifs 13; CIDP, chronic inflammatory demyelinating polyneuropathy; CNTN1, contactin 1; Caspr1,2, contactin associated protein1,2; Dsg1,3, desmoglein 1, 3; DPPX, dipeptidyl peptidase-like protein 6; FAE, Fab-arm exchange; GSH, glutathione; Iglon5, IgLON family member 5; Lgi1, leucine-rich, glioma-inactivated 1; Lrp4, low-density lipoprotein receptor-related protein 4; MBL, mannose-binding lectin; MuSK, muscle-specific kinase; MG, myasthenia gravis; PLA2R, phospholipase A2 receptor; THSD7A, thrombospondin type-1 domain-containing 7A; TTP, thrombotic thrombocytopenic purpura; vWF, von Willebrand factor.
